# The Texas Medical Association

**Published:** 1886-06-20

**Authors:** 


### THE TEXAS MEDICAL ASSOCIATION

met at Dallas on 21st April last. The meeting was a large and successful one. Mayor Brown welcomed the Association in behalf of the city. Dr. Thurston, from Dallas County Medical Association, responded in appropriate terms.

Dr. E. L. Belton, President of the Association, then addressed the body touching upon the delinquency of members, the International Medical Congress, sustaining the action of the American Medical Association. He spoke also in opposition to asking legislation in behalf of the medical profession, holding that educated physicians need no protection, and that quacks cannot be suppressed by legal enactment. Any effort made in this direction will be misconstrued and treated with contempt.

Many interesting papers were presented to the Association.

Dr. Salem, of Austin, was expelled from the body "for conduct unbecoming a gentleman and practitioner."

Dr. Stout took strong ground against specialists in the profession.

Dr. Farrar exhibited a rare pathological specimen—a liver weighing nineteen pounds and seven ounces.

Dr. Stailey reported a case of lithotomy, and showed a stone two and three-fourth inches long and four and a half inches in circumference. The patient recovered.

The following officers were elected for the ensuing year, to-wit:

President—Dr. Thos. H. Mott, of Goliad.

1st Vice-President—Dr. Chelton, of Dallas.

2d Vice-President—Dr. Loggins, of Ennis.

3rd Vice-President—Dr. Parsons, of Terrell.

The city of Austin was selected as the next place of meeting.

STARTLING DISCLOSURE.—A few days ago the managing editor received by mail a small tin box snugly wrapped and sealed. On opening the same he was startled by the appearance therein of a living, active, moving creature, having horns, claws and tail. Visions of centipeds, tarantulas, snakes, scorpions, dynamite and infernal machines flashed across his mind. It was truly a horrible object to behold, especially to one who had not before seen an animal of the kind. The package had not been heralded by any notice or forerunner of warning, thus allowing full force to the shock of such an unlooked for and horrid apparition, and no name of the party to whom we are indebted for this startling surprise accompanied the package. Only the following words were found on a slip within the box—"Texas ant-eater or horned frog."

"OLD FRIEND, CLASS OF 1884."

---

QUESTION OF VERACITY BETWEEN DR. BATTEY AND LAWSON TAIT.—We note that Mr. Lawson Tait now claims to have first operated for the Removal of Normal Ovaries on August 1, 1872, while Battey first operated on August 17, 1872. Battey, in referring to the matter, plainly intimates that Mr. Tait has economized the truth. Perhaps Battey thinks that Tait is seeking to *emasculate* him of his well earned honors, and wou'd say to him *out-cast-trater*.

